# Menopausal symptoms in relationship to breast cancer-specific quality of life after adjuvant cytotoxic treatment in young breast cancer survivors

**DOI:** 10.1186/s12955-020-1283-x

**Published:** 2020-02-10

**Authors:** Winnie Yeo, Elizabeth Pang, Giok S. Liem, Joyce J. S. Suen, Rita Y. W. Ng, Christopher C. H. Yip, Leung Li, Claudia H. W. Yip, Frankie K. F. Mo

**Affiliations:** 1 Department of Clinical Oncology, Prince of Wales Hospital, Sir YK Pao Centre for Cancer, Faculty of Medicine, The Chinese University of Hong Kong, Prince of Wales Hospital, NT, Hong Kong, Hong Kong Special Administrative Region China; 2 Hong Kong Cancer Institute, State Key Laboratory of Translational Oncology, Faculty of Medicine, The Chinese University of Hong Kong, Hong Kong, Hong Kong Special Administrative Region China

**Keywords:** Cytotoxic, MENQOL, FACT-B + 4, QoL

## Abstract

**Introductions:**

For young premenopausal breast cancer patients, adjuvant chemotherapy may cause menstrual disruptions and premature menopause, which may in turn impair their quality of life (QoL). In this study among young breast cancer survivors who have undergone adjuvant chemotherapy, the objectives were to assess post-treatment menopausal symptoms and their associated factors, and to correlate these symptoms with breast cancer-specific QoL.

**Methods:**

The study population included premenopausal young Chinese women with early-stage breast cancer who had undergone adjuvant chemotherapy between 3 and 10 years prior to enrolling into this study. At study entry, patients’ characteristics and clinical features were collected; each patient had detail menstrual history collected and each filled in MENQOL and FACT-B + 4 questionnaires.

**Results:**

Two hundred eighty eligible patients were recruited. For adjuvant chemotherapy, 92% received anthracyclines and 28% received taxanes; 76% received adjuvant tamoxifen. At a median of 5.0 years from initial cancer diagnosis, 49 and 11% had become post- and peri-menopausal respectively. MENQOL at study entry revealed that physical domain score was worse in overweight/obese patients (mean scores for underweight/normal vs overweight/obese: 2.65 vs 2.97, *p* = 0.0162). Vasomotor domain score was worse in those who received taxanes or tamoxifen (taxane vs non-taxane: 2.91 vs. 2.35, *p* = 0.0140; tamoxifen vs no tamoxifen: 2.75 vs. 2.34, *p* = 0.0479). Sexual domain score was worse among those who had become peri/post-menopausal (peri/postmenopausal vs premenopausal: 2.82 vs. 2.29, *p* = 0.0229). On the other hand, patients who utilized traditional Chinese medicine had significantly worse scores for vasomotor, psychosocial and physical domains. Further, there was a significant association between MENQOL scores and FACT-B + 4 scores; less severe symptoms in the MENQOL domains were associated with better QoL scores in FACT-B + 4 physical, functional, psychosocial and emotional well-being, Breast Cancer Subscale, Arm Subscale and FACT-B total score.

**Conclusion:**

Among premenopausal breast cancer women who had undergone adjuvant chemotherapy, those who had received taxanes or tamoxifen, were overweight/obese and utilized traditional Chinese medicine had more severe menopausal symptoms. Patients who experienced worse menopausal symptoms were found to have worse breast cancer-specific QoL. Interventional studies with an aim to alleviate menopausal symptoms are warranted to assess if overall QoL of these patients could be improved.

**Trial registration:**

Not applicable.

## Introduction

Breast cancer is one of the most common female malignancies. In many parts of Asia, there has been an increasing trend in the incidence of breast cancer. The most recent data from the Hong Kong Cancer Registry have reported that more than 80% of newly diagnosed breast cancer patients have early stage disease [1]. Treatments for patients with early-stage breast cancer have curative intent. These mainly involve surgery followed by post-operative adjuvant therapies that may include chemotherapy, radiotherapy, endocrine therapy and/or targeted therapy. Although less than 15% of patients were reported to be diagnosed before age 40 [2], a higher proportion of these patients were subjected to adjuvant cytotoxic therapy [2].

Anti-neoplastic therapies, in particular those that involve cytotoxic agents, are associated with short- as well as long-term adverse effects [3–13]. Particularly among premenopausal women, the drug’s effect on ovarian function could potentially lead to long-term menstrual disruptions. Our recent report on premenopausal young breast cancer survivors who were subjected to adjuvant cytotoxic therapy have noted that over 90% developed chemotherapy-related amenorrhea (CRA) within the first year of chemotherapy, with 50% developing chemotherapy-related menopause, in which over 60% reached menopause before aged 45 [14]. Patients who had undergone adjuvant chemotherapy have been reported to experience physical, emotional and social changes that may affect their quality of life (QoL) [15–19]. Whilst earlier studies have suggested that the deterioration in QoL after chemotherapy may be associated with the transition of menopause [15, 16, 18], there has been limited data that demonstrate the association of menopausal symptoms on the overall QoL in young breast cancer survivors who have received adjuvant cytotoxic chemotherapy. Deciphering the relationship of menopausal symptoms and QoL may enable clinicians to better inform and advise patients prior to their anti-cancer therapies; furthermore, such knowledge is also valuable for researchers to explore potential interventions that could increase patients’ ability to cope with their symptoms and thereby improve their QoL.

In this study, young premenopausal Chinese breast cancer survivors who had undergone adjuvant chemotherapy were assessed. The objectives were to assess the symptoms related to menopause and the associated factors, as well as to determine the association of these symptoms with breast cancer-specific QoL during follow-up.

## Patients and methods

The study enrollment took place between September 2008 and February 2011 in the Department of Clinical Oncology of Prince of Wales Hospital, Hong Kong. Consecutive breast cancer patients attending the breast cancer follow-up clinics, who were identified to be eligible for the study, were invited to participate in the study. Inclusion criteria included premenopausal Chinese females who were diagnosed with early-stage (stage I to III) breast cancer at age 45 years or less and had received adjuvant chemotherapy. At study entry, these patients should have been between 3 and 10 years from their initial breast cancer diagnosis. Patients who had undertaken hysterectomy prior to breast cancer diagnosis and those who had undergone medical, surgical or radiation oophorectomies were excluded. The investigators obtained written informed consent from each individual prior to study entry. At study entry, patients were asked by a study nurse on the details of their menstruation history. Following this, patients filled in self-administered questionnaires to assess their menopausal-specific as well as breast cancer-specific QoL using Menopause-Specific Quality of Life Questionnaire (MENQOL) and Functional Assessment of Cancer Therapy–Breast (FACT-B + 4) questionnaires respectively. The study was approved by the Joint CUHK-NTEC Clinical Research Ethics Committee of the Hong Kong Hospital Authority and the Chinese University of Hong Kong.

### Data collection of clinical information

Patients’ demographics were collected. Their clinical records were retrieved for breast tumor characteristics and the details of their anti-cancer treatments.

### QOL assessment using menopause-specific quality of life questionnaire (MENQOL)

MENQOL is a validated menopause-specific QoL instrument that consists of 29 items which assesses the impact of four domains of menopausal symptoms, namely, vasomotor (three items), psychosocial (seven items), physical (sixteen items) and sexual (three items). A validated Chinese version was used [20]. Each item is scored from 0 (not bothersome) to 6 (extremely bothersome). For analyses, the item scores are further converted to a score ranging from 1 to 8 by adding 1 or 2, depending on whether the individual does or does not experience the item, respectively. The mean of the domain is used as the overall domain score; with each domain score ranging from 1 to 8, with higher scores reflecting worse QoL [21].

### Breast cancer-specific QOL assessment using functional assessment of Cancer therapy-breast (FACT-B + 4)

FACT-B + 4 is a breast cancer-specific multidimensional QoL assessment instrument. FACT-B + 4 version consists of 41 items which are divided into six subscales assessing physical well-being, emotional well-being, social well-being, functional well-being, Breast Cancer Subscale and Arm Subscale. A validated Chinese version was used [22]. Each item is rated on a 5-point Likert scale. Negatively worded items are recoded such that a higher score indicates a better QOL. Scores from physical well-being, emotional well-being, social well-being, functional well-being and Breast Cancer Subscale yield a FACT-B total score, with higher scores reflecting better QoL [23, 24].

The study questionnaires were distributed to patients who agreed and consented to participate in the study. During the study, a research nurse or assistant conducted cross-check of each study questionnaire that individual patients had filled in; patients who were identified to have incomplete questionnaires were then asked to fill in all the missing parts in the same visit.

### Sample size estimation

Based on historical data, the rate of CRA in western breast cancer patient ranged from 50 to 80%. The study assumed the rate of CRA in the studied population to be equivalent to the western population and that a 20% difference in this rate would be of no clinical significance. If the true rate was 0.7 with alpha level equal to 0.05, and assuming that the rate of CRA in western breast cancer patients was 0.6, then the required sample size for having a 90% power would be 260. With an expected 10% lose due to patient refusal or administrative error, the total number to be recruited was targeted at 290.

### Statistical analysis

The study applied SAS version 9.3 for statistical analysis. Data on clinical characteristics were summarized as patient number (n) and percentage (%) for categorical variables, and mean with standard deviation for continuous variables.

The t-test or ANOVA test for mean comparison was performed to identify any factors associated with impairment in MENQOL. In addition, the correlation between MENQOL and FACT-B + 4 was examined. The MENQOL domains with mean cut-off were used to compare different FACT-B + 4 subscales. Statistical analyses were two-sided, with *p*-value < 0.05 being regarded as significant.

## Results

### Patients’ background demographics and breast cancer characteristics

Consent was sought from all eligible patients; 95% (286 patients) agreed to partake in the study. Of these, 6 patients were subsequently found to be ineligible, thus resulting in 280 eligible patients participating in the study. Table 1 shows the patients’ background characteristics and clinical features. At breast cancer diagnosis, the median age was 41 years. For adjuvant cytotoxic chemotherapy, 92.1% had received anthracyclines and 28.2% had received taxanes. Two hundred and fourteen patients had received tamoxifen adjuvant therapy; 53.3% were still on the treatment at study entry.

**Table 1:** Patients’ characteristics and clinical features (*n* = 280)

	No. of patients	%
Age at time of breast cancer diagnosis		
≤ 35	42	15
36–40	81	28.9
41–45	157	56.1
Age at study entry		
≤ 35	8	2.9
36–40	26	9.2
41–45	76	27.1
46–50	146	52.1
> 50	23	8.6
Education		
Primary	47	16.9
Secondary	187	67.0
Tertiary and higher qualification	45	16.2
Marital Status		
Single	43	15.4
Married Partner	216	77.1
Divorced	15	5.4
Widowed	6	2.1
Family income*		
< HK$5000	27	9.6
HK$5000-25,000	150	53.6
> HK$25,000	103	36.8
BMI at study entry (kg/m^2^) [25]		
< 18.5 (Underweight)	11	3.9
18.5–22.9 (Normal)	123	44.0
23.0–24.9 (Overweight)	62	22.1
> 25 (Obese)	84	30.0
Staging		
I	88	31.4
II	165	58.9
III	27	9.6
Hormonal receptor		
ER positive	203	72.5
PR positive	187	66.8
HER overexpression	47	16.8
Breast surgery:		
Lumpectomy	95	33.9
Mastectomy	185	66.1
Axillary nodal dissection	276	98.6
Adjuvant radiotherapy	186	66.4
Adjuvant chemotherapy regimen:		
Taxane-containing	79	28.2
Non-taxane-containing	201	71.8
Adjuvant chemotherapy lasting longer than 64 days	191	68.2
Time since last adjuvant treatment**		
3 to < 5 years	160	57.1
5 to < 10 years	120	42.9
Ever received adjuvant tamoxifen	214	76.4
On adjuvant tamoxifen at study entry	115	41.1
Adjuvant targeted therapy with trastuzumab	8	2.9
Utilization of traditional Chinese medicine since diagnosis	83	29.6
Menopausal status at study entry		
Premenopausal	112	40
Peri/Post- menopausal	168	60

*HK$ = Hong Kong dollars; HK$1 is equivalent to US$ 0.128

** Only include chemotherapy/radiotherapy or trastuzumab

The median interval between breast cancer diagnosis and study entry was 5.0 years (range: 3.0–9.9). At study entry, the median age of the studied population was 46.5 years (range: 28–54). One hundred and twelve patients (40%) remained premenopausal, 137 (49%) were post-menopausal while 31 (11%) were found to be peri-menopausal.

### Analysis for MENQOL at study entry

The mean scores and standard deviation for vasomotor, psychosocial, physical and sexual domains, as well as the proportions of patients scoring lower versus those scoring equal to or above the mean are listed in Table 2.

**Table 2:** Mean scores of the four domains in MENQOL (n = 280)

MENQOL domain	Mean (SD)	No. of patients with scores < mean (%)	No. of patients with scores >/= mean (%)
Vasomotor	2.51 (1.70)	177 (63.2)	103 (36.8)
Psychosoical	2.76 (1.41)	161 (57.5%)	119 (42.5%)
Physical	2.82 (1.10)	150 (53.6%)	130 (46.4%)
Sexual	2.61 (1.90)	173 (61.8%)	107 (38.2%)

Outcomes on analysis of potential factors that could be associated with MENQOL are listed in Table 3. Worse vasomotor domain scores occurred in patients who received taxane-containing chemotherapy (taxane- vs. non-taxane containing regimens: 2.91 vs. 2.35, *p* = 0.0140), those who received tamoxifen (tamoxifen vs. no tamoxifen, 2.75 vs. 2.34, *p* = 0.0479) and those who adopted traditional Chinese medicine (TCM; yes vs. no: 2.94 vs. 2.33, *p* = 0.0054). Patients who utilized TCM also scored worse for psychosocial domain (yes vs. no: 3.14 vs. 2.59, *p* = 0.0028) and physical domain (yes vs. no: 3.14 vs. 2.70, *p* = 0.0052). Patients who were overweight/obese scored worse for physical domain (underweight/normal vs overweight/obese: 2.62 vs. 2.97, *p* = 0.0162). Patients who were peri−/post- menopausal at study entry had worse scores for sexual domain (premenopausal vs peri/post-menopausal: 2.29 vs. 2.82, *p* = 0.0229).

**Table 3:** MENQOL scores based on the four domains among studied patients

	vasomotor domain			psychosocial domain			physical domain			sexual domain		
	Mean score	SD	*p*	Mean score	SD	*p*	Mean score	SD	*p*	Mean score	SD	*p*
Overall population	2.51	1.70	–	2.76	1.41	–	2.82	1.10	–	2.61	1.90	–
No. of patients with scores >/= mean (%)	103 (36.8)			119 (42.5)			130 (46.4)			107 (38.2)		
Age at time of study entry			0.1227			0.1036			0.6317			0.2397
≤ 40	2.25	1.69		2.28	1.13		2.73	1.14		1.99	1.53	
41–45	2.46	1.73		2.93	1.51		2.95	1.20		2.65	1.84	
46–50	2.70	1.75		2.81	1.41		2.78	1.03		2.70	2.02	
> 50	1.90	1.01		2.48	1.34		2.74	1.13		2.81	1.70	
BMI at study entry			0.8753			0.3717			**0.0162**			0.2760
Underweight or Normal	2.53	1.73		2.68	1.37		2.65	1.07		2.74	1.93	
Overweight or Obese	2.50	1.67		2.83	1.44		2.97	1.11		2.49	1.87	
Breast surgery:			0.9321			0.6877			0.5563			0.1375
Lumpectomy	2.52	1.69		2.71	1.42		2.76	1.05		2.37	1.64	
Mastectomy	2.50	1.70		2.78	1.41		2.84	1.13		2.73	2.01	
Axillary nodal dissection			0.6127			0.3630			0.7797			0.1500
Yes	2.52	1.70		2.75	1.41		2.81	1.10		2.63	1.90	
No	2.08	1.95		3.39	0.88		2.97	0.61		1.25	0.50	
Adjuvant radiotherapy			0.7467			0.3781			0.7246			0.9861
Yes	2.53	1.58		2.70	1.36		2.83	1.05		2.61	1.87	
No	2.46	1.76		2.86	1.50		2.78	1.18		2.61	0.50	
Adjuvant chemotherapy regimen:			**0.0140**			0.9373			0.7131			0.9094
Taxane-containing	2.91	1.89		2.75	1.26		2.85	1.02		2.59	1.84	
Non-taxane-containing	2.35	1.59		2.76	1.46		2.80	1.13		2.62	1.92	
Duration of adjuvant chemotherapy			0.2232			0.3966			0.8625			0.8946
< =64 days	2.33	1.75		2.86	1.51		2.80	1.14		2.63	2.09	
> 64 days	2.60	1.57		2.71	1.36		2.82	1.08		2.60	1.80	
Time since last adjuvant treatment*			0.2568			0.4249			0.3966			0.3051
3 to < 5 years	2.61	1.69		2.70	1.36		2.77	1.08		2.51	1.85	
5 to < 10 years	2.38	1.70		2.83	1.47		2.88	1.13		2.74	1.96	
Ever received adjuvant tamoxifen			0.8659			0.8076			0.8320			0.7338
Yes	2.52	1.76		2.77	1.38		2.82	1.12		2.59	1.88	
No	2.48	1.50		2.72	1.52		2.79	1.03		2.68	1.98	
On adjuvant tamoxifen at study entry			**0.0479**			0.7057			0.9349			0.6175
Yes	2.75	1.84		2.79	1.32		2.82	1.10		2.54	1.86	
No	2.34	1.57		2.73	1.47		2.81	1.10		2.65	1.93	
Adjuvant targeted therapy with trastuzumab			0.7117			0.8083			0.9443			0.7262
Yes	2.29	1.20		2.88	1.56		2.79	0.72		2.38	1.86	
No	2.52	1.71		2.75	1.41		2.82	1.11		2.61	1.90	
Utilization of traditional Chinese medicine since diagnosis			**0.0054**			**0.0028**			**0.0052**			0.3732
Yes	2.94	1.80		3.14	1.55		3.10	1.19		2.76	1.95	
No	2.33	1.62		2.59	1.31		2.70	1.04		2.54	1.88	
Menopausal status at study entry			0.2079			0.7446			0.9338			**0.0229**
Premenopausal	2.35	1.67		2.72	1.49		2.82	1.16		2.29	1.64	
Peri/Post- menopausal	2.62	1.71		2.78	1.35		2.81	1.06		2.82	2.03	

*Only include chemotherapy/radiotherapy or trastuzumab

### Analysis on FACT-B + 4 score in association with MENQOL domain scores at study entry

Details of the outcome of FACT-B + 4 have been described in a recent report [18]. For the present analysis, the mean scores and standard deviation for FACT-B + 4 subscales, and those of breast cancer subscale, arm subscale and FACT-B total score are listed in Table 4.

**Table 4:** Fact-B score for all patients with breast cancer in association with MENQOL domain scores

FACT-B Subscales	Overall population	MENQOL vasomotor domain		MENQOL psychosocial domain		MENQOL physical domain		MENQOL sexual domain	
		< Mean	>/= Mean	< Mean	>/= Mean	< Mean	>/= Mean	< Mean	>/= Mean
Physical well-being	23.4	24.5	21.5	25.0	21.2	25.2	21.3	23.8	22.6
SD	4.14	3.73	4.19	3.11	4.35	2.88	4.36	4.10	4.10
*p*-value	–	< 0.0001		< 0.0001		< 0.0001		0.0152	
Functional well-being	19.9	20.5	18.8	21.8	17.4	21.5	18.0	20.9	18.3
SD	5.45	5.67	4.87	5.22	4.71	5.26	5.06	5.02	5.77
*p*-value	–	0.0100		< 0.0001		< 0.0001		0.0001	
Psychosocial well-being	19.4	19.9	18.6	20.6	17.7	20.7	17.9	20.0	18.5
SD	6.03	5.98	6.08	6.05	5.61	5.80	5.98	5.99	6.02
*p*-value		0.1034		< 0.0001		0.0001		0.0479	
Emotional well-being	17.7	18.4	16.5	19.3	15.5	19.1	16.0	18.5	16.3
SD	4.19	3.82	4.54	3.39	4.16	3.49	4.32	4.09	4.02
*p*-value	–	0.002		< 0.0001		< 0.0001		< 0.0001	
BCS score	22.3	23.7	19.8	24.3	19.5	24.1	20.1	23.2	20.6
SD	5.26	5.16	4.42	4.85	4.44	5.28	4.33	5.07	5.19
*p*-value	–	< 0.0001		< 0.0001		< 0.0001		< 0.0001	
Arm subscale	14.9	15.7	13.5	16.2	13.1	16.4	13.2	15.5	13.9
SD	3.92	3.68	3.95	3.41	3.86	3.23	3.94	3.83	3.87
*p*-value	–	< 0.0001		< 0.0001		< 0.0001		0.0005	
FACT-B total score	102.6	107.0	95.2	111.1	91.2	110.7	93.3	106.4	96.5
SD	18.78	18.28	17.30	15.73	16.44	16.20	17.21	17.92	18.58
*p*-value	–	< 0.0001		< 0.0001		< 0.0001		< 0.0001	

Analysis on association between MENQOL scores and FACT-B + 4 scores showed that, in general, patients with lower scores (less symptoms) for MENQOL domains had significantly better QOL in terms of FACT-B + 4 subscales (Table 4). For instance, when compared with patients who had lower than mean vasomotor domain score, those with scores equal to or higher than the mean had worse FACT-B + 4 physical well-being (24.5 vs. 21.5 respectively, *p* < 0.0001), functional well-being (20.5 vs. 18.8 respectively, *p* = 0.01), emotional well-being (18.4 vs. 16.5 respectively, *p* = 0.002), BCS score (23.7 vs. 19.8 respectively, *p* < 0.0001), Arm subscale (15.7 vs. 13.5 respectively, *p* < 0.0001) and FACT-B total score (107.0 vs. 95.2 respectively, *p* < 0.0001). When comparing patients who had lower than mean MENQOL psychosocial domain score, those with scores equal to or higher than the mean had worse FACT-B + 4 physical well-being (25.0 vs. 21.2 respectively, *p* < 0.0001), functional well-being (21.8 vs. 17.4 respectively, *p* < 0.0001), psychosocial well-being (20.6 vs. 17.7 respectively, *p* < 0.0001), emotional well-being (19.3 vs. 15.5 respectively, *p* < 0.0001), BCS score (24.3 vs. 19.5 respectively, *p* < 0.0001), arm subscale (16.2 vs. 13.1 respectively, *p* < 0.0001) and FACT-B total score (111.1 vs. 91.2 respectively. Similar findings were also observed for MENQOL physical domain and sexual domain, where lower scores for these domains were significantly associated with better QOL in terms of FACT-B + 4 physical well-being, functional well-being, psychosocial well-being, emotional well-being, BCS scores, Arm subscale and FACT-B total scores.

## Discussion

Quality of life (QoL) among breast cancer survivors may be affected by the stage of the disease and the treatment required. It is well-acknowledged that breast cancer patients with metastatic disease suffer most from symptom burden and this is associated with worsening QoL [26]. On the other hand, it is also increasingly recognized that women with early-stage disease also experienced impairment in QoL [26]. Management of breast cancer patients have traditionally focused on effectiveness of anti-cancer treatment, such that in the case of patients with early disease, cure from breast cancer has been the key objective. With advances in adjuvant treatments, the survival of breast cancer patients has continuously improved during the past decades. However, with longer survival, side effects of anti-cancer treatments, especially those of long-term sequelae, have become increasingly apparent and this has been associated with adverse effects on QoL. In the present study on young Chinese breast cancer survivors who had previously undergone adjuvant chemotherapy, the objectives were to assess chemotherapy-associated symptoms that were related to long-term menstrual disturbances and to evaluate their effect on breast cancer-specific QoL. Findings from the current study revealed that five years after breast cancer diagnosis, women who had received taxanes and tamoxifen, those who were overweight/obese and those who utilized traditional Chinese medicine had worse menopausal symptoms. Further, patients who experienced worse menopausal symptoms were found to have worse breast cancer-specific QoL.

In premenopausal breast cancer patients who have received chemotherapy, endocrine changes may affect fertility and menstrual status, which in turn may have a detrimental effect on QoL [26–28]. The decrease in estrogen secondary to ovarian failure is marked by a series of vasomotor symptoms including night sweats and hot flashes along with other symptoms such as vaginal dryness, dyspareunia and weight changes [28]. When compared to women who had undergone natural menopause, breast cancer patients who developed menopause following anti-cancer treatment have reported more severe menopausal symptoms [28].

Few studies have formally assessed menopausal symptoms in relation to quality of life after adjuvant cytotoxic chemotherapy. An Australian study that involved 843 breast cancer survivors had assessed menopausal symptoms using MENQOL during follow-up of up to 5 years after initial diagnosis. The result revealed that, in comparison to women enrolled from the community who did not have history of breast cancer, breast cancer survivors had significantly worse scores for vasomotor and sexual domains. The impact on QoL may differ according to pre-treatment menopausal status. In the Australian study, breast cancer survivors who were postmenopausal were more likely to report on vasomotor and sexual symptoms than their premenopausal counterpart. However, findings from this study was limited by a low representation of premenopausal women, with pre- or peri- menopausal patients accounting for only 7% of the whole studied population, and assessment on menopausal symptoms in relation to chemotherapy was only confined to 40% of the overall studied population who had undergone adjuvant chemotherapy [29]. In another study that was conducted in Korea, which addressed issues on menopausal symptoms among premenopausal patients who had undergone chemotherapy, MENQOL was reported to be persistently worse after adjuvant chemotherapy; however, any changes beyond the first year were not assessed [30]. Risk analysis revealed that older age was associated with worse symptoms in all the four (vasomotor, psychosocial, physical and sexual) domains, tamoxifen usage was associated with worse symptoms in vasomotor, physical and sexual domains, while a BMI of >/= 23 kg/m^2^ was associated with worse physical symptoms [30].

The present study was undertaken to specifically assess a homogenous group of young premenopausal Chinese young breast cancer patients who had received adjuvant chemotherapy; with their endocrine-related symptoms and the associated factors were investigated, while long-term breast cancer-specific QoL issues in relation to menopausal symptoms were evaluated. Data from the present study highlights that patients who underwent taxane-containing chemotherapy and those who received tamoxifen had worse vasomotor symptoms. Tamoxifen has been well-associated with vasomotor adverse effects, while taxane-containing regimen has been reported in a number of studies to be associated with increased incidences of chemotherapy-related amenorrhea [8, 10–12, 30]. However, as taxanes are often given following anthracycline and cyclophosphamide-containing chemotherapy, it is difficult to ascertain whether the agents per se, or whether the extended duration of cytotoxic regimens that involve taxanes, leads to the higher incidence of amenorrhea [9], which in turn, could lead to increased vasomotor symptoms. Sexual symptoms were worse among patients who became peri- or post-menopausal after chemotherapy. This lends support to findings from previous studies that assessed premenopausal breast cancer patients, where sexual interest and functioning were noted to be worst among those who received chemotherapy and subsequently became amenorrheic after treatment [31]. Patients who utilized TCM also scored worse for vasomotor, psychosocial and physical domains. The causal relationship of TCM with menopausal symptoms remains to be clarified. TCM is often adopted among Chinese patients in an attempt to alleviate menopausal symptoms. Indeed, interventional studies have suggested the potential efficacy of pharmacological and non-pharmacological maneuvers as well as complementary medicine in relieving side effects including that of vasomotor symptoms due to anti-cancer therapy [33–34]. When MENQOL was assessed together with outcomes of FACT-B + 4, patients who had worse menopausal symptoms were associated with poorer breast cancer-specific QoL in all aspects, reflecting a wide dimension of QoL impairment with the occurrence of menopausal symptoms.

Findings from the present study are limited by the fact that menopausal symptoms and QoL assessments were only conducted at one time-point without longitudinal follow-up and that the patient number was limited. Another limitation of the study was the lack of exploration of the effect of potential confounding factors on the reported associations by the construction of a linear regression model. Nonetheless, the current study provides a snapshot into the close association between endocrine-related symptoms and breast cancer-specific QoL experienced by young Chinese breast cancer survivors after adjuvant chemotherapy. With increasing knowledge on long-term adverse effects of cytotoxic chemotherapy, clinicians could potentially support patients by better preparing them for their anti-cancer therapies. At the same time, researches into interventional therapies that tackle specific menopausal symptoms which are bothering and affecting the QoL of patients are needed. Interventions that have been advocated include pharmacological as well as non-pharmacological means [35]. However, it has to be noted that to date, interventional therapies that have been suggested have not been confirmed to be effective in large scale clinical trials. As such, high quality interventional studies with a focus on young breast cancer patients are required to provide evidence-based clinical recommendations to clinicians, so that appropriate and efficacious management for menopausal symptoms can be offered in order to make a positive impact on their QoL.

## Conclusion

In this study among premenopausal Chinese women with breast cancer who had undergone adjuvant chemotherapy, having received taxanes or tamoxifen, being overweight/obese and having utilized TCM were associated with more severe menopausal symptoms. The current study demonstrates that the multifactorial nature of menopausal symptoms has an impact on QoL. Further studies using serial assessments in a larger sample of patients would enable a better understanding of the dynamic changes of these aspects in relation to timing of chemotherapy and other adjuvant therapies.

## Data Availability

All data based on this study has been included in the manuscript.

## Abbreviations

FACT-B + 4: Functional Assessment of Cancer Therapy-Breast
MENQOL QOL: Menopause-Specific Quality of Life Questionnaire
n: patient number
QoL: quality of life
TCM: traditional Chinese medicine
